# Intestinal Excessive Activity

**Published:** 1910-10

**Authors:** G. M. Saleba

**Affiliations:** Roanoke, Ala.


### INTESTINAL EXCESSIVE ACTIVITY. PHYSIOLOGICAL CONSIDERATION.

By G. M. Saleba, M. D., D. D. S., Roanoke, Ala.

As the chyme passes along the small intestine, the chyle and other soluble constituents are absorbed, and what remains is moved onward into the great intestine, where it forms the bulk of the feces.

Along the whole route, fluid is passing in both directions between the intestinal contents and the blood from the bowel into the vessel and from the vessel into the bowel.

The consistency of the feces, will, therefore, depend upon the activity of absorption, the activity of excretion, and manifestly, the rate of transit. The more active the absorption, the less active the excretion, and the slower the rate of transit, so much the firmer will be the feces; whilst liquidity of the feces will be the result of imperfect absorption, excessive excretion, or rapid transmission.

We are accustomed to speak of the one extreme as constipation, and of the other as diarrhoea.

Absorption from the bowel is carried on by the lacteal and portal system, and depends upon the state of the organs in which it is accomplished, as well as the nature of the chemical constituents of their contents, excretion is rather active in th small intestine. The transit is effected by paristalsis. The muscular coat is inverted by the vagus which increases paristalsis, while the splanchnics tends to restrain, or inhibit it. With this physiological touch we will now consider.———Pathological Relations: As far as our present purpose is concerned the disturbances of the intestines independently of its digestive function, is chiefly excessive action, the striking phenomenon of which is diarrhoea.

The term diarrhoea, indicative of increased motor activity or of increased, or, preverted secretory activity in the intestinal canal; and is generally preferable to gastric or duodenal dyspepsia.

It has been said that diarrhoea is a symptom and not a disease, and that the disease of which it is a symptom, is inflammation of the intestinal mucous membrane, or intestinal catarrh. From the view point of treatment, this question is not, perhaps, of much importance, and we may regard diarrhoea both as a symptom and a disease.

Certainly there are forms of diarrhoea which are not inflammatory, and cannot be rightly considered as dependent on inflammation of the intestinal mucous membrane, and in which this so-called symptom is of almost sole importance, and it may, therefore, be justly regarded as a functional disease. We consider, that there is justification for the division of diarrhoea into inflammatory and non-inflammatory. The latter including those cases of emotional origin, or nervous diarrhoea, analogous to the polyurea of a hysterical attack, and those of mere exaggeration of physiological function, habitual in some persons, and in many instances dependent on any slight excess in the quantity of the ingesta. In those cases, too, the matters discharged are ordinary faecal matters, simply in an unduly liquid state. In cases of diarrhoea dependnt on inflammatory catarrh of the intestine, the flux from the bowel consists in part of the inflammatory exudation from the catarrhal mucous membrane, in part of intestinal secretion hurried onward by the exaggerated paristalsis attendant on inflammation, and in part of unabsorbed partially or wholly undigested food. In chronic diarrhoea, due to chronic intestinal catarrh, abundant muco-purulent secretion may be present in the discharges.

With these few preliminary remarks, we may pass on to the consideration of the causes of acute and chronic diarrhea.

(1) Those referable to gastric or duodenal dyspepsia.

(2) Certain intestinal irritants generated in the body itself.

(3) Disorders of the general and abdominal circulation.

THE MOST COMMON CAUSES.

(1) The most common cause of intestinal hyperaemia, catarrh and diarrhea is local irritation, and the most usual local irritant is some offending ingesta. A variety of food substances will, under certain conditions, excited diarrhoea, either substances unsuitable in quality, i. e., in themselves indigestible, and acting as irritating foreign bodies, or substances in a state of commencing decomposition, and capable of acting as chemical irritants, or simply food too copious in quantity, or badly prepared. The pouring out of an excess of bile, or bile of an afnormal quality, may act in the same way, or on the other hand, an absence of bile from inflammatory closure of the common duct, may also lead to diarrhoea, for in absence of the alkaline bile, which usually neutralises the acidity of the chyme as it passes out of the stomach, the contents of the small intestines remain acid, and, therefore, act as an irritant, and set up an intestinal catarrh from the lack of bile. The loose motions are in such cases found to be white or clay colored, and often very offensive, as a due admixture with bile also prevents the formation of irritating products of putrefaction by its known antiseptic action.

Long retained indurated faeces may set up catarrhal inflammation of the intestine, not only by mechanical irritation, but also by the toxic substances developed by the putrefactive changes they undergo.

(2) The influence of cold is now recognized to be a much more common cause of intestinal catarrh than it used to be. It seems more likely that the catarrh so caused is dependent either on a reflex vaso-motor influence arising through the cutaneous nerves, that it is dependent on arrested cutaneous secretion causing the retention in the blood of substances irritating to the intestinal mucous membrane.

(3) The ntestnal catarrh may be secondary, and due to a morbid state of the intestinal mucous membrane, which may itself be caused by some other organic, or, constitutional disease, e. g., obstruction to the circulation through the liver or in the portal vein, causing venous hyperaemia of the intestine or a more general and more permanent venous congestion dependent on chronic pulmonary and cardiac disease.

(4) Many instances of endemic or epidemic diarrhoea, such as are specially prone to occur in hot weather and probably of microbic origin, and the elevation of temperature, which has been regarded as a cause of diarrhoea, may only act by calling the infactive organism into activity, or by promoting the activity of putrefactive processes in foods and beverages. Under this heading we should group those cases which occur after drinking water from suspected sources, and probably contaminated with sewage matters, and those associated with malarial infection. The green diarrhoea of infants has been shown to be of microbic origin.

(5) Some attacks of diarrhoea are eliminative and are excited by the presence of toxic substances in the blood, as in uraemia, etc.

(6) Infancy seems to be a predisposing cause of intestinal catarrh, which is very common in nursing children. This is due not only to the extreme sensitiveness of their intestinal mucous membrane, but also to the frequent absence of that extreme care and cleanliness which are needed in the selection and preparation of their food in order to avoid exciting gastro-intestinal irritation. The too early recourse to farinaceous foods is especially responsible for much of the gastro-intestinal disorders of infancy.

There have been, and exist today so many theories as to the cause of summer complaints of children that time will not permit me to take them up, but there is one theory above all others that I desire to see eliminated from the minds of every physician and layman; this is the teething theory. This theory has been instilled into the minds of not only the physicians, but also every other human being on earth, and I believe it is this theory alone that is responsible for the large death rate of infants and children.

As a heated term is almost upon us, and judging the future by the past, hundreds of children will be carried to their graves, I take pleasure in presenting to you for discussion the treatment of the summer complaints of children.

We have nearly as many remedies as there are stars in heaven, every physician and old granny have their favorites. My idea is, the fewer remedies used, the better results will be obtained. In the short time I am allowed to read this paper, I will only be able to take up the principal points of treatment and not go into detail, though this is a very important factor.

The chief points in the treatment of summer complaints of children whether it may be gastro-intestinal, entero-colitis, or colitis, are four:

(1) To assist the effort which nature is making to free the stomach and intestine from the poison which is in them.

(2) To restore the surface circulation which is so seriously interfered with.

(3) To supply water to the tissues which are being drained to so great an extent.

(4) To support the strength of the patient until the disease has run its course.

When diarrhoea exists, the digestive functions fail to act, therefore the best results are obtained by abstinence from food for twenty of twenty-four hours. The stomach and bowel should be properly cleansed by lavage if possible, and by a laxative as calomel, or often better still, by castor oil. For twenty-four or forty-eight hours, sterilized water only should be given or a little rice water or arrow-root, tapioca, or sago cooked with water. When given to older children these drinks may be flavored with nutmeg, cloves or cinnamon. This food should be sterile and fed from bottles or dishes scrupulously clean. In most cases it is necessary to abandon the use of milk for at least two or three days——often for a longer time. As a rule, milk cannot be tolerated because the casein in it is not well digested. During this time it is best to give very little food—not more than a tablespoon or two every one or two hours. Beside the ice water or other farinaceous waters just mentioned, a little beef tea, or veal or mutton broth may be given.

Egg albumen stirred into water may also be used. Children one or two years old can take the cereal waters best and after the first days a somewhat thicker gruel. When improvement begins and it seems necessary to give more nourishing food, a modified milk containing 3% or fat, 4 or 5% of sugar, and 2 or 3% of protied may be employed. The mixture should be made slightly alkaline by the addition of lime water.

As digestion becomes well established, the ingredients of the milk may be made to approach nearer and nearer to the normal proportion of cow's milk or mother's milk to which the child has been accustomed.

Flushing the colon with hot sterile salt water with or without castile soap, is of great importance. It should be done once or twice daily and in some cases even more often. A large quantity of water, not less than a gallon, should be used so that the colon will be well cleansed at least in the lower parts. This not only helps to cleanse the intestine, but also helps to restore by absorption to the blood some of the fluid that has been lost, to soothe the intestine and to warm the little patient's body.

After the stomach has been thoroughly cleansed with warm water containing a spoonful of common salt to the pint, small doses of calomel should be given dry on the tongue. If vomiting returns, rejeat the irigation. Of course the calomel is given for its anti-fermentative action, and in order to reach the small intestines, which are inaccessible by the process of irrigation. If rectal temperature is very high, ice cold water can be given by enemata. When the vomiting has continued for sometime and there is prostration with great thirst, the infant should be allowed to suck sterilized ice-cold water from the bottle. If vomiting and diarrhoea continue to be excessive, a hypodermic of morphine 1-100 grain and atropine 1-800 grain should be given to an infant a year old. The effect should be carefully watched, and the dose repeated if necessary.

To restore the circulation or if there are signs of collapse, nothing is better than the hot pack. This should be done by wrapping the patient to the chin in sheets wrung out of water at least at hot as 100 F., and should be then enveloped in a hot blanket. This procedure should be repeated as often as necessary. While the infant is in the pack, water can be given freely by the mouth, and a subcutaneous injection of normal salt solution should be given.

In some cases, a thin starch solution to which a few minims of laudanum have been added may be gently thrown into the rectum, to be repeated if necessary. For intestinal putrefaction nothing is better than salol and salicylate or subnitrate of bismuth, though every physician has a favorite prescription. To relieve the straining, to lessen the frequency and to change the character of the stools, nothing acts better than the following: R Magnesian sulphat grs. 5; Tr. opii deodorized gtt ½ to 1; Syr. tolu Min 15; Aq. memth pipp. q. s. dram 1. M. Sig. To be repeated every two, three or four hours if necessary, for a child one year old. The opium should be ordered separately, as one might want to increase or decrease the dose, or leave it out entirely.

During convalescence tonics are indicated. It is a help to give such children a change of air, especially to get them upon the water, where fresh, clean, invigorating air will blow over them.

Anodynes, antiseptics, astringents, and stimulants have their places in the treatment, but will surely fail unless hygienic and dietetic rules are also followed.

Before closing, I beg to emphasize that so long as dentition and atmospheric influences are not causative factors as it used to be supposed, Prophylaxis now should demand a careful consideration in these cases. It is true up to now we are not able to demonstrate the idea of contagion in summer diarrhoea of children, but prophylactic methods will tend, "as the weight of authority has demonstrated" to lessen the spread of these diseases.

It will be useless for me to dwell with emphasis on the subject of prophylaxis, as the cry of the modern age is strong for better sanitary methods, and hundreds are now enlisted in this great movement. The medical man by force of ability should be the leader in such a noble undertaking. Medical science has proved by recent researches in thousands of instances that medicinal treatment is not the prime factor, but abortive treatment as well as hygienic methods are of great importance.

Holt emphasizes prophylactic means in the following manner:

(1) In sending children out of unhealthy locations to the open air and the fields.

(2) In education of the laity as to the importance of regularity in feeding, and the great danger of over-feeding as well as the proper preparation of food.

(3) Proper legal regulation as to the examination of milk.

(4) The exclusion of germs from all foods given, especially in milk, by sterilization, and by cleasing bottles, nipples, and all materials that may come in touch with the mouths of children.

(5) Prompt attention to all mild disarrangements.

(6) Cutting down the amount of food, and increasing the amount of water during the excessive summer heat.

Read before C. V. M. and S. A. Roanoke, Ala., July 12th., 1910.

